# Eye lymphatic defects induced by bone morphogenetic protein 9 deficiency have no functional consequences on intraocular pressure

**DOI:** 10.1038/s41598-020-71877-z

**Published:** 2020-09-29

**Authors:** Mariela Subileau, Niyazi Acar, Alison Carret, Lionel Bretillon, Isabelle Vilgrain, Sabine Bailly, Daniel Vittet

**Affiliations:** 1 University of Grenoble Alpes, Inserm, CEA, IRIG-DS-BCI, 38000 Grenoble, France; 2 Centre des Sciences du Goût et de l’Alimentation, AgroSup Dijon, CNRS, INRAE, Université Bourgogne Franche-Comté, 21000 Dijon, France

**Keywords:** Eye diseases, Ocular hypertension, Glaucoma, Lymphangiogenesis

## Abstract

Aqueous humor drainage is essential for the regulation of intraocular pressure (IOP), a major risk factor for glaucoma. The Schlemm’s canal and the non-conventional uveoscleral pathway are known to drain aqueous humor from the eye anterior chamber. It has recently been reported that lymphatic vessels are involved in this process, and that the Schlemm’s canal responds to some lymphatic regulators. We have previously shown a critical role for bone morphogenetic protein 9 (BMP9) in lymphatic vessel maturation and valve formation, with repercussions in drainage efficiency. Here, we imaged eye lymphatic vessels and analyzed the consequences of *Bmp9* (*Gdf2*) gene invalidation. A network of lymphatic vessel hyaluronan receptor 1 (LYVE-1)-positive lymphatic vessels was observed in the corneolimbus and the conjunctiva. In contrast, LYVE-1-positive cells present in the ciliary bodies were belonging to the macrophage lineage. Although enlarged conjunctival lymphatic trunks and a reduced valve number were observed in *Bmp9*-KO mice, there were no morphological differences in the Schlemm’s canal compared to wild type animals. Moreover, there were no functional consequences on IOP in both basal control conditions and after laser-induced ocular hypertonia. Thus, the BMP9-activated signaling pathway does not constitute a wise target for new glaucoma therapeutic strategies.

## Introduction

Glaucoma is a group of neuropathies associated with retinal ganglion cells and optic nerve degeneration that can lead to severe vision loss. Primary open-angle glaucoma (POAG) is the prevalent form of these diseases. Elevated intraocular pressure (IOP) appears as a major risk factor for POAG and is currently the main therapeutic target in the treatment of this disease^1^. High IOP may result from drainage defects and/or from increased resistance to aqueous humor outflow from the anterior chamber of the eye. The Schlemm’s canal, connecting the anterior chamber to the venous vascular system, is the major aqueous humor drainage pathway^1^. In this pathway, called conventional, the aqueous humor flows through the trabecular meshwork into the lumen of the Schlemm’s canal that drains by the intermediary of collector channels into the episcleral blood vessels. Aqueous humor can also exit from the anterior chamber through the interstitial spaces of the ciliary muscles to the supraciliary and the suprachoroidal spaces, before being returned back to the venous circulation^2,3^. This route, called non-conventional, has been postulated to also involve the lymphatic vessel network, suggesting the existence of an uveolymphatic drainage pathway^4,5^. In addition to important functions for immune cell trafficking, it is established that lymphatic vessels play an essential role in maintaining interstitial tissue fluid homeostasis^6^.

Lymphatic vessels are present in the eye corneolimbus and in the superficial eye conjunctiva^7,8^. Some studies have also postulated for the existence of lymphatics in more internal parts of the eye such as the ciliary body and/or the choroid, but these statements remain controversial and need to be further confirmed^9–11^. Lymphatics of the anterior segment of the eye appeared to be potentially involved in the drainage of aqueous humor. Pioneer work from Pr McMenamin’s group reported the accumulation of fluorescent antigens into cervical lymph nodes after prior intracamerular delivery into the eye anterior chamber^12^. More recent studies from Dr Guignier’s team^13^ and from Pr Yücel’s group^14–17^ have brought additional evidences for an aqueous humor outflow by an uveoscleral lymphatic pathway component. These authors showed that intracamerally injected tracers could diffuse and accumulate into mandibular and/or cervical lymph nodes, supporting a putative lymphatic aqueous humor drainage route. Consistent with this assumption, Latanoprost was shown to raise the ocular lymphatic drainage in mice, leading to a decrease in IOP^16^. Prostaglandin F2 alpha analogs, such as Latanoprost, are potent anti-hypertensive medication in glaucoma that primarily increase aqueous humor outflow via the non-conventional uveoscleral pathway, and also partly through the conventional outflow facilities^18–20^. Following binding to prostaglandin FP receptors, their effects were described to be mainly related with the rapid induced relaxation of both ciliary muscle cells and trabecular meshwork cells, and further extracellular matrix remodeling after metalloproteinases synthesis. Another more recent work also mentioned that Latanoprost have reduced IOP-lowering effects in patients who have undergone cervical lymph node surgery^21^. Overall, these observations support the hypothesis of the involvement of a lymphatic component in aqueous humor outflow regulation.

The Schlemm’s canal, constitutes a major component of the conventional aqueous humor outflow pathway^22^. It originates from blood vessels at postnatal developmental stages^23^. Several works have established that the Schlemm’s canal shares several properties with lymphatic vessels^23–26^. Indeed, Schlemm’s canal endothelial cells express several lymphatic markers, and some molecular signaling pathways regulating the lymphatic system are critical for its formation. One of the most characterized is the angiopoietin/tie2 (ANGPT/TIE2) signaling pathway (known as ANGPT/TEK in human), which is a major regulator of vascular development. Several studies performed with genetically modified mutant mice have established its importance in eye lymphatic vessels and Schlemm’s canal development and maintenance with strong effects on IOP^27–29^. The significant role of this pathway was further established in patients by the finding that some mutations in the ANGPT/TEK signaling are the cause of primary congenital glaucoma^28,30^. On the other hand, the vascular endothelial growth factor C / vascular endothelial growth factor receptor 3 (VEGF-C/VEGFR3) ligand/receptor system, a main regulatory pathway for lymphatic development and lymphangiogenesis was also reported to be involved in Schlemm’s canal development in mice and to exert significant effects in the IOP levels^25^.

Bone morphogenetic protein 9 (BMP9), also called Growth differentiation factor 2 (GDF2), is a circulating protein that binds with high affinity to activin receptor-like kinase 1 (ALK1), a type I receptor of the TGFβ receptor family, that is important for blood vascular development^31^. BMP9, which is detected as soon as E10 in the mouse embryo^32^, and which is highly expressed from early postnatal to adult stages^33^, was shown to be secreted by the liver, considered to be the prominent source of the cytokine^33,34^. Moreover, it was found to be present at active concentrations in blood^33^, and can act at distant targets sites for physiological processes regulation. Among its actions, the BMP9/ALK1 signaling pathway was reported critical pathway for the development and maturation of lymphatic vessels. Indeed, targeting either BMP9 or its type I receptor ALK1 in neonate mice resulted in abnormal lymphatic remodeling in several organs, including the skin, the mesentery and the diaphragm^35–37^. BMP9 was also identified as essential for lymphatic valve formation^37^. Interestingly, the consequences of *Bmp9* gene invalidation in the mouse were still observed in the skin and the diaphragm of adult mice in parallel of a reduced lymphatic drainage efficiency in limb^37^.

In this context, we thus wondered whether the BMP9/ALK1 signaling pathway was also involved in the development of eye lymphatic vessels and Schlemm’s canal, further displaying some repercussions on IOP. To answer these questions, our major goals were: (1) to characterize the distribution of lymphatics in the mouse eye; (2) to determine the consequences of *Bmp9* gene invalidation on Schlemm’s canal and eye lymphatic vessels architecture, and (3) to measure whether functional consequences are observed on IOP in the absence of BMP9. This information may be relevant in order to know whether targeting BMP9/ALK1 signaling pathway could be useful for the development of new therapeutic strategies for glaucoma.

## Results

### Visualization and characterization of the eye surface lymphatic vessel network.

**Figure 1:**
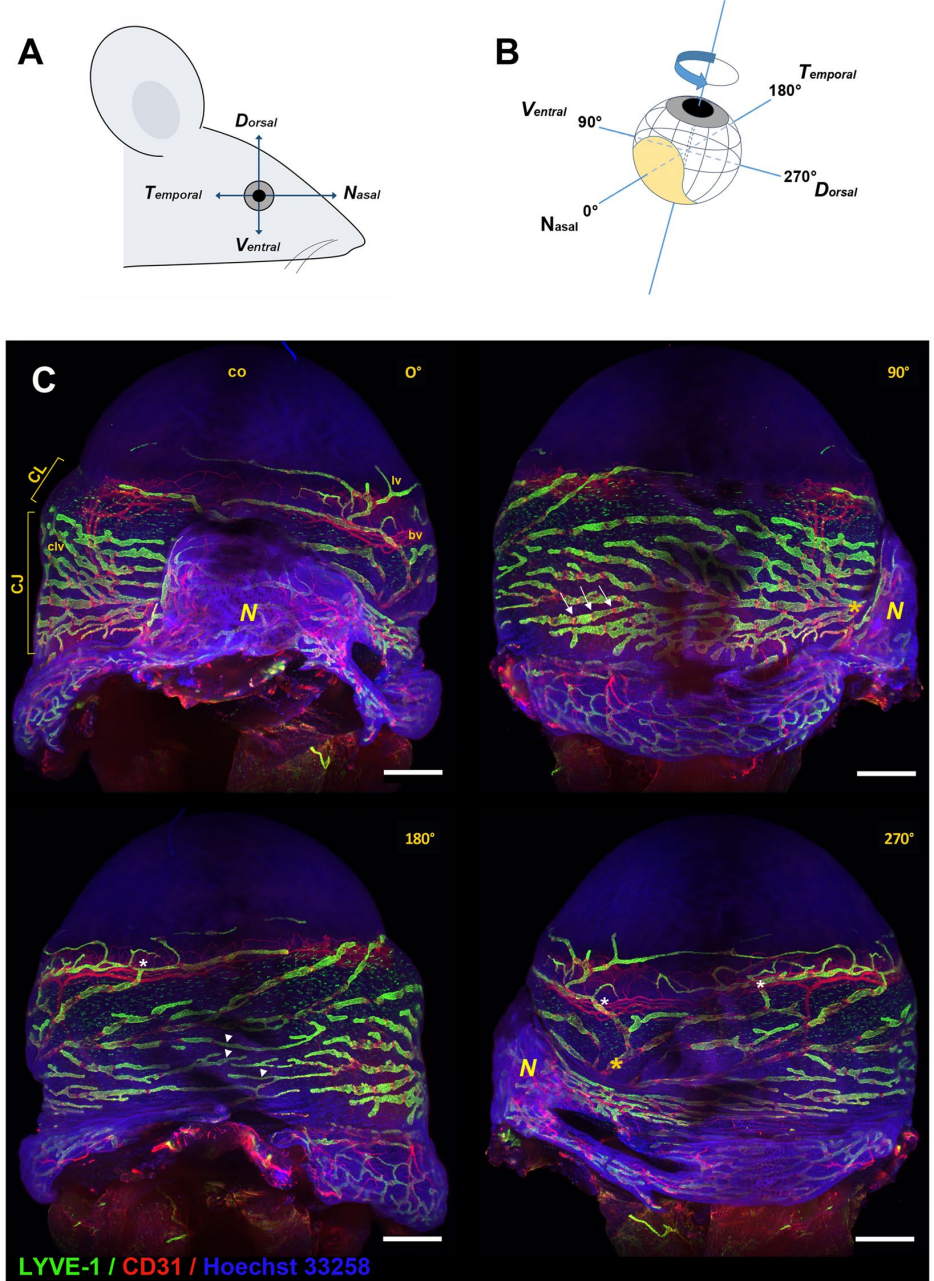
Right eye lymphatic vessel network imaging by light sheet fluorescence microscopy (LSFM). (**A**) View of the cardinal axes of the mouse eye. (**B**) Schematic representation of the multiview angle sequence used for the image acquisition and of the position of the images shown. The nictictating membrane (N) is drawn in yellow. (**C**) Whole mount immunofluorescence stainings of the eye were performed with LYVE-1 (green) and CD31 (red) antibodies. Nuclei counterstaining was performed with Hoechst 33258 (blue). Multi-angle LSFM images were acquired in order to visualize the whole surface of the eyeball. The eye was imaged sequentially at four different projections, as indicated (0°, 90°, 180° and 270° rotation around the vertical axis). The corneolimbus (CL) displays a ring of both LYVE-1-positive lymphatic vessels (lv) and CD31-positive blood vessels (bv) encircling the basis of the avascular cornea (co). A rich network of conjunctival lymphatic vessels (clv) is seen in the underneath bulbar conjunctiva (CJ). White arrows pointed to some CD31-positive/LYVE-1-negative areas corresponding to valve location. *N*: nictitating membrane. White asterisks indicated some connection points of the main corneolimbal lymphatic vessel with the lymphatic conjunctival network, whereas yellow asterisks pointed to the position of the two lymphatic collecting trunks draining either the ventral part (lymphatics left to the nictitating membrane; 90° projection image) or the dorsal part (lymphatics right to the nictitating membrane; 270° projection image) of the eyeball. Some thinner lymphatic vessels located at the opposite side of the nictitating membrane (180° projection image) are pointed by white arrowheads. Scale bars: 500 µm.

In this study, we first aimed at further characterizing the distribution and the organization of the mouse adult eye lymphatic network. Imaging of the whole topography of the surface vessel network covering the eye was performed by Light Sheet Fluorescence Microscopy (LSFM) technology. Using lymphatic vessel hyaluronan receptor 1 (LYVE-1), an antigenic marker of lymphatic endothelial cells, and CD31, a pan-endothelial cell antigenic marker expressed on both lymphatic and blood endothelial cells, we observed the presence of a rich lymphatic vascular network on the external surface of the eye. As expected, LYVE-1-positive lymphatic vessels were found in the corneolimbus and the conjunctiva (Fig. 1). Corneolimbal lymphatic vessels exhibiting ramifications and some small extensions were encircling the limbus at the border of the avascular cornea. These LYVE-1-positive corneolimbal lymphatics appeared to be connected and to flow into the lymphatic conjunctival network located underneath and covering the entire surface of the bulbar conjunctiva. Interestingly, the multiview imaging highlighted a heterogeneous but characteristic conjunctival lymphatic vessel distribution around the eyeball. Two main networks draining either the dorsal part or the ventral part of the eyeball exited through two large lymphatic trunks on both sides of the translucent nictitating membrane (also called *plica semilunaris* in mammals) at the nasal side of the eye (Fig. 1). These two networks were located in mirror in relation to the position of the mouse nose. For the right eye, the lymphatic network located in the dorsal side of the eyeball drained into the trunk located at the right side of the nictitating membrane whereas the denser and more ramified network that drained the lower ventral part of the conjunctiva exited at the left side of the nictitating membrane. Moreover, the thinner lymphatic vessels appeared to be located in the temporal side at the opposite of the nictitating membrane (Fig. 1). This vessel distribution was symmetrically opposite when considering the left eye (Supplementary Fig. S1). The LSFM images also showed that the LYVE-1-positive lymphatic vessels of the conjunctiva had some blind-ended extremities and that they contained several intraluminal valves depicted here in red, as valve-forming cells were LYVE-1-negative and CD31-positive. A heterogeneity was also observed for the corneolimbal lymphatics which were connected to the underneath conjunctival lymphatic vessel network. On the other hand, perilimbal blood vessels were visualized by their single CD31^high^-positive red labeling, since they lack LYVE-1 expression.

**Figure 2:**
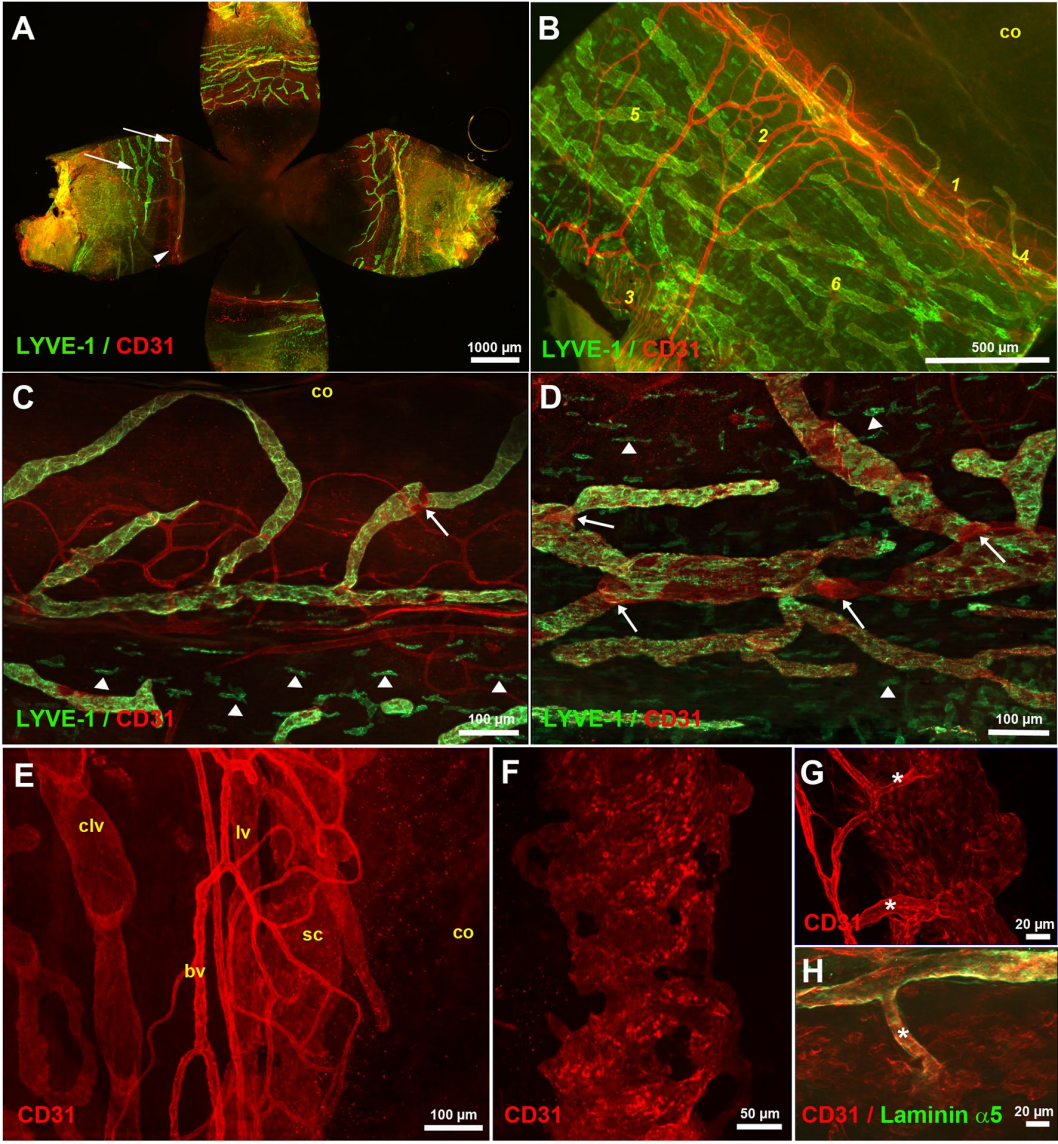
Whole mount LYVE-1 and CD31 immunostainings of the dissected eye anterior segment. (**A**) Imaging of the eye four quadrants after flat-mounting. The corneolimbal and the dense conjunctival lymphatic vessel networks are revealed by the green LYVE-1 immunostaining (arrows). Some CD31-positive blood vessels (red) displaying a low staining intensity are also detected in the iridocorneal region (arrowhead). (**B**) Morphological appearance of the eye superficial vasculature on a quadrant. Perilimbal (*1*) and episcleral (*2*) blood vessels that further associate with the extraocular muscle vasculature (*3*) are visualized by the CD31 immunostaining. LYVE-1 stains the main corneolimbal lymphatic vessel (*4*) with some extensions at the border of the cornea (co), and the rich network of lymphatics of the conjunctiva (*5*). LYVE-1-negative CD31-positive areas correspond to lymphatic valves (*6*). (**C**,**D**) High magnifications of the corneolimbal region (**C**), and of a representative part of the conjunctiva (**D**). Some of the single LYVE-1-positive cells that align parallel with the lymphatic vessels are pointed by white arrowheads. The white arrows indicate the location of valves. *co*, cornea. (**E**) View of the whole mount CD31 immunostaining of the iridicorneal angle region. The Schlemm’s canal (sc) appears below the blood (bv) and lymphatic (lv) corneolimbal vasculature. One can note that a conjunctival lymphatic vessel (clv) exhibiting valves is present on the left of the image. *co*, cornea. (**F**) Schlemm’s canal morphology revealed by CD31 immunostaining. Projection image of the z-stack corresponding to the Schemm’s canal depth. (**G**,**H**) Details of the CD31 (**G**) and CD31 and laminin α5 (**H**) immunostainings of collector channels connecting the Schlemm’s canal to a blood vessel. Collector channels are indicated by asterisks.

To get more insights into the distribution of eye lymphatic vessels, we also considered whole mount immunostainings of the dissected eye anterior segment (Fig. 2). The results of these experiments are in accordance with our previous observations (Fig. 2A,B). Corneolimbal and conjunctival lymphatic vessels were revealed by their LYVE-1-positive expression. The lymphatic valve location was visualized by the LYVE-1-negative and high CD31 expression of the cells constituting the valve territories (Fig. 2B–D). On the other hand, a high number of single LYVE-1-positive cells was detected in the limbus and in the conjunctiva (Fig. 2C,D). These cells, which may correspond to previously described LYVE-1-positive macrophages^38^, were differently organized according to the tissue layers of the eyeball surface. They were found orientated either in longitudinal lines paralleled to corneolimbal and conjunctival lymphatic vessels (Fig. 2C,D), or were aligned in more vertical lines, perpendicular to the corneolimbal vessels, in more deep cell layers (Supplementary Fig. S2). The imaging of the iridocorneal region allowed the visualization of the Schlemm’s canal located underneath the corneolimbal vessels. Although the Schlemm’s canal exhibited several lymphatic properties, it did not express LYVE-1 antigen and was therefore visualized by its CD31-positive staining. As observed at a higher magnification of the corneolimbal region, the Schlemm’s canal appeared as a large channel running below the main corneolimbal lymphatics and the loops of the blood vessels (Fig. 2E). The z-stack projection of the acquired images specifically corresponding to the Schlemm’s canal revealed its large and flatten characteristic structure with an irregular shape as well as the presence of holes (Fig. 2F). Several collector channels connecting the Schlemm’s canal to the venous blood vessels could be seen (Fig. 2G). Interestingly, they did not exhibit any evidence for the presence of valves preventing the backflow of blood into the Schlemm’s canal. Indeed, no valve displaying CD31 stained leaflets with a core matrix of laminin α5, such as described in classical lymphatic valves^39^, could be seen at the connection of the collector channels with the Schlemm’s canal (Fig. 2H). Although the eye lymphatic surface network was easily imaged, the presence of black pigmented epithelia and of melanocytes in the C57BL/6 mice was associated to limitations in signal penetration through deep tissues and precluded the obtaining of information on the existence and the distribution of lymphatic vessels in the internal eye uvea.

### Analysis of the presence of lymphatics in ciliary bodies.

**Figure 3:**
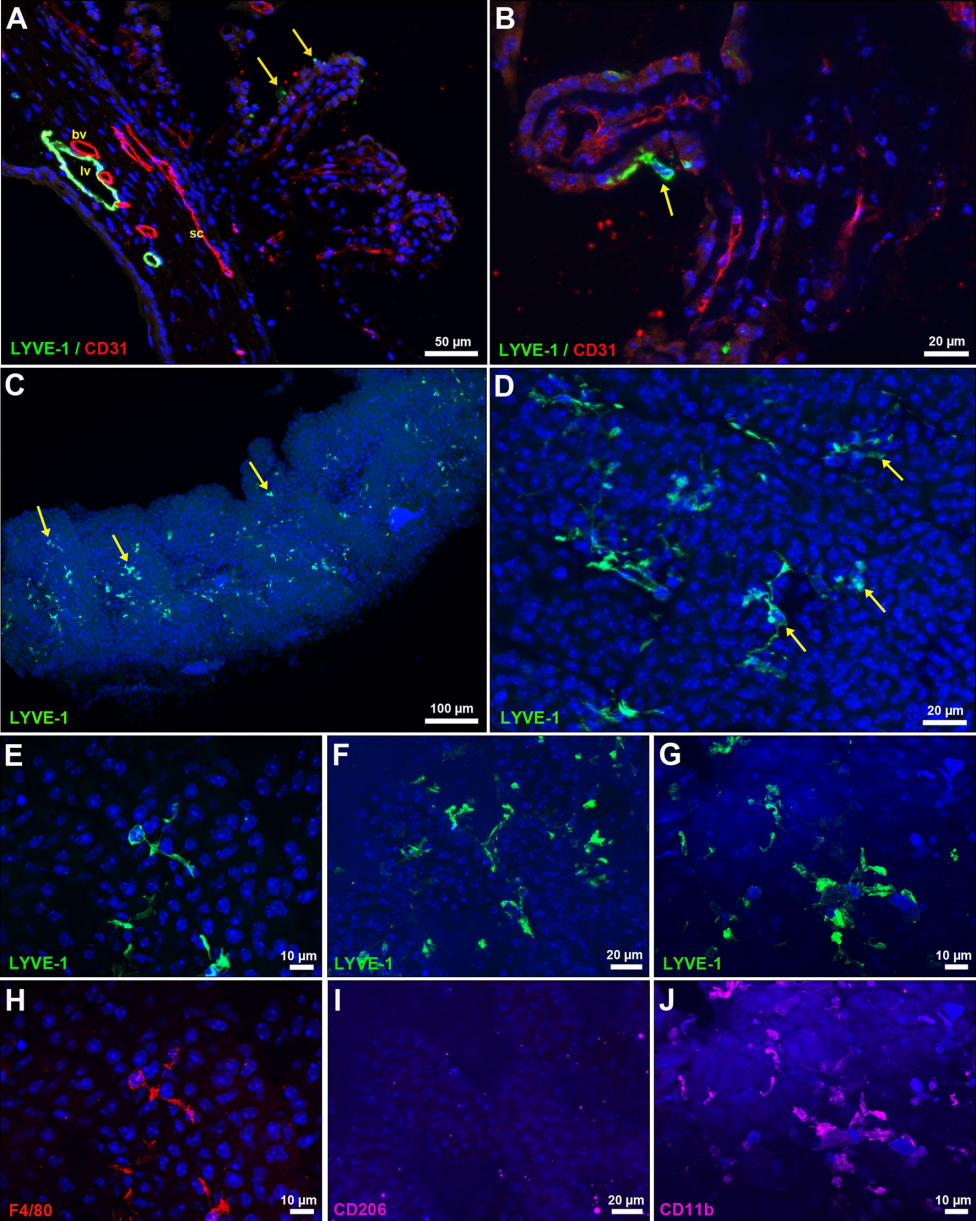
Analysis of the presence of lymphatics in the eye internal uveal tract. (**A**) Cryosection immunostainings with CD31 (red) and LYVE-1 (green) antibodies of the iridocorneal angle. Arrows point to some LYVE-1-positive spots at the surface of a ciliary process. *sc*, Schlemm’s canal; *lv*, lymphatic vessel; *bv*, blood vessel. (**B**) High magnification corresponding to the staining of a cryosection of the ciliary processes. The arrow indicates a LYVE-1-positive cell. (**C**–**J**) Whole mount immunostainings of ciliary processes after melanin depigmentation by H_2_O_2_ treatment. (**C**,**D**) LYVE-1 expressions in ciliary processes. Arrows pointed to some single LYVE-1-positive cells. (**E**–**J**) Analysis of the expression of the antigenic macrophage markers F4/80, CD206 and CD11b by the LYVE-1-positive cells in ciliary processes. All images are projection from z-stacks acquired with AxioImager fluorescence microscope equipped with an apotome.

In order to further image lymphatics in more internal eye structures and since the presence of lymphatics involved in aqueous humor drainage has been postulated into the ciliary bodies^14^, we performed CD31 and LYVE-1 immunostainings on eye cryosections. This allowed the visualization of blood vessels, Schlemm’s canal and lymphatics at the iridocorneal angle (Fig. 3A). In addition to the LYVE-1-positive vessels of the corneolimbus, frozen eye anterior segment sections also revealed some small LYVE-1-positive spots on the surface of ciliary processes (Fig. 3A). The observed labelling was mostly consistent with the image of a section through dispersed cellular bodies (Fig. 3B). To get a better insight on this staining, we then performed whole mount immunofluorescent stainings combined with further eye depigmentation by H_2_0_2_ treatment^40^. The imaging of dissected ciliary bodies revealed the presence of many single LYVE-1-positive cells (Fig. 3C). These cells displayed ameboid and/or microglial/macrophage-like cell shapes, and were mainly distributed at the surface of the ciliary processes without showing any evidence for structures evoking lymphatic vessels (Fig. 3D–G). Further analysis of the expression of three macrophage antigenic markers, differentially expressed by M1 and M2 macrophage subtypes, confirmed that these cells belonged to the myeloid/macrophage lineage and that they were non-endothelial cells. Indeed, LYVE-1-positive cells present in the ciliary processes, were F4/80- and CD11b-positive and displayed low or negative CD206 expression (Fig. 3E–J). Moreover, as indicated above, a large number of single LYVE-1-positive cells exhibiting macrophage-like morphology were also seen in the limbus and the conjunctiva, some of them being observed in a close proximity with vessels. In contrast with what was observed in the ciliary processes, most of them were positive for CD206 and CD11b but not for F4/80 (Supplementary Fig. S3).

### Consequences of *Bmp9* gene invalidation on Schlemm’s canal and eye lymphatic vessels.

**Figure 4:**
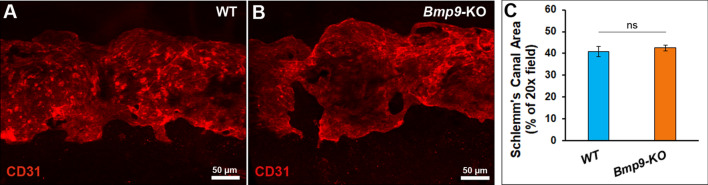
Comparative analysis of Schlemm’s canal in eyes of WT and *Bmp9*-KO mice. (**A**,**B**) Representative images of Schlemm’s canal morphology in WT and *Bmp9*-KO mice after whole mount CD31 immunostaining. (**C**) Quantitative analysis of the Schlemm’s canal area between WT and *Bmp9*-KO mice. Measurements of CD31-positive areas were done using image J software on at least 3 images at ×20 magnification per eye. Values are the means ± SEM from 11 eyes from 6 WT mice and from 15 eyes from 8 *Bmp9*-KO mice. *ns*, not significant.

As we previously reported that BMP9 was involved in lymphatic vessel development and maturation in several organs^37^, we then investigated whether *Bmp9* gene invalidation in mice had an effect on Schlemm’s canal morphogenesis and/or on eye lymphatics. We have given priority to the study of aged mice since the prevalence of glaucoma and aqueous humor drainage defects are predominantly observed in the elderly human patients^1^. We found that *Bmp9*-KO mice displayed normal Schlemm’s canal morphogenesis compared to WT mice (Fig. 4A–C). Similar findings were also observed at early developmental time points (P7 and P19) and for young adults aged 2–3 months old (Supplementary Fig. S4). These observations indicated that BMP9 was not critical for Schlemm’s canal development and maintenance.

**Figure 5:**
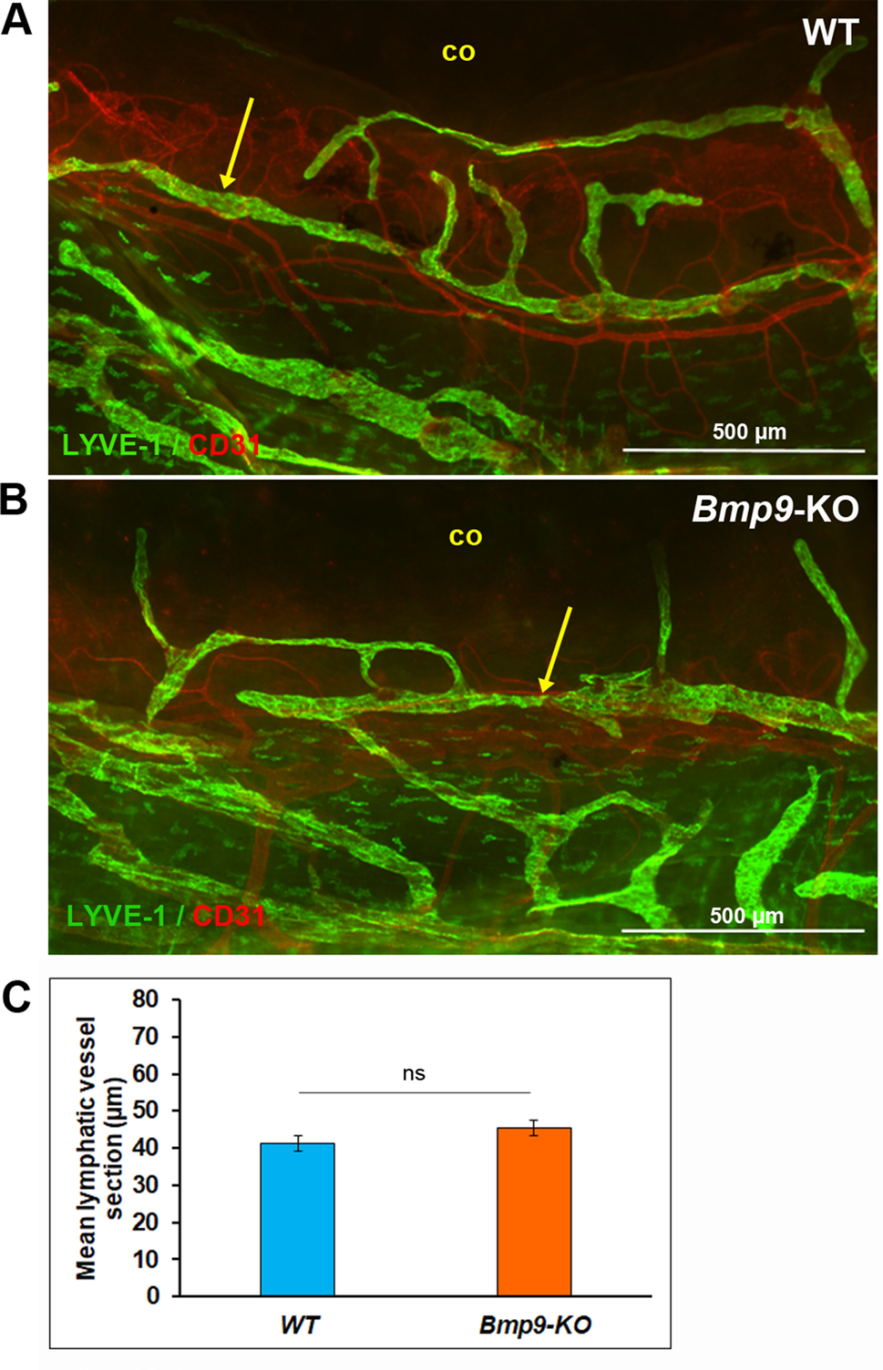
Comparative analysis of eye corneolimbal lymphatic vessels in WT and *Bmp9*-KO mice. (**A**,**B**) Representative images of LYVE-1 (green) and CD31 (red) immunostainings of the corneolimbal vessels in WT (**A**) and *Bmp9*-KO (**B**) mice. The yellow arrows point to the main corneolimbal lymphatic vessel that encircles the avascular cornea (co). (**C**) Quantitative analysis of the mean lymphatic vessel section on the image of the LYVE-1 staining. Values are the means ± SEM from 13 eyes from 8 WT mice and from 20 eyes from 11 *Bmp9*-KO mice. *ns*, not significant.

**Figure 6:**
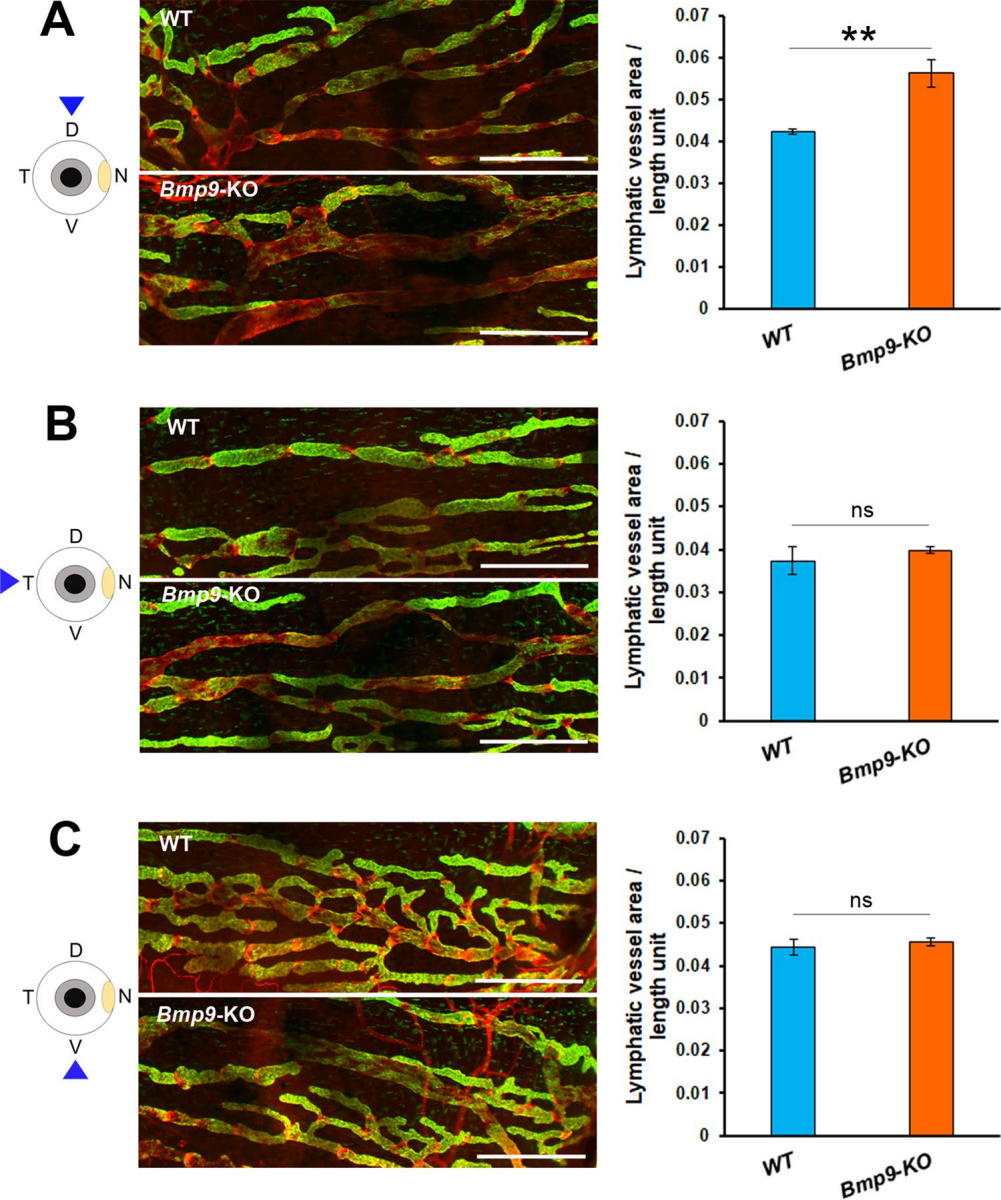
Comparative analysis of eye conjunctival lymphatic vessels in WT and *Bmp9*-KO mice. Left panels: representative images of conjunctival lymphatic vessels (LYVE-1 (green) and CD31 (red) immunostainings) in right eyes of WT and *Bmp9*-KO mice. Image facing up the dorsal (**A**), the temporal (**B**) or the ventral (**C**) side of the eye, as indicated by the corresponding drawings at the left of the figure. Right panels: quantitative analysis of the mean lymphatic vessel area per length unit of lymphatic vessel on the images of the different regions of the conjunctiva. Values are the means ± SEM from 5 eyes for both WT and *Bmp9*-KO mice. **p < 0.01, significantly different from WT by Mann–Whitney *U* test. Scale bars: 500 µm.

With regard to lymphatic vessels, since we did not get any evidence for the existence of conventional lymphatics in ciliary bodies, we focused our attention on the morphology of the lymphatic corneolimbal and conjunctival networks. Again, we have examined aged adult mice for the analysis. The morphological appearance of corneolimbal LYVE-1-positive lymphatic vessels was similar in the two genotypes (Fig. 5A,B). Accordingly, the measurements of vessel sections, performed on the images of the LYVE-1 immunostainings, provided similar results and did not reveal any significant difference (Fig. 5C). When considering the conjunctival lymphatic vasculature, an enlargement in the collecting trunks draining the dorsal conjunctival network was noticed in *Bmp9*-KO mice (Fig. 6A). In contrast, the morphometric analysis did not reveal any significant difference for the thinner lymphatics located at the opposite side of the nictitating membrane and for the lymphatic vessels draining the ventral region of the conjunctiva (Fig. 6B,C). In parallel, we still observed within the *Bmp9*-KO mice group, enlarged lymphatic capillaries displaying increased vessel diameter in the trachea, used as a control of the persistence of the *Bmp9*-KO phenotype (Supplementary Fig. S5).

**Figure 7:**
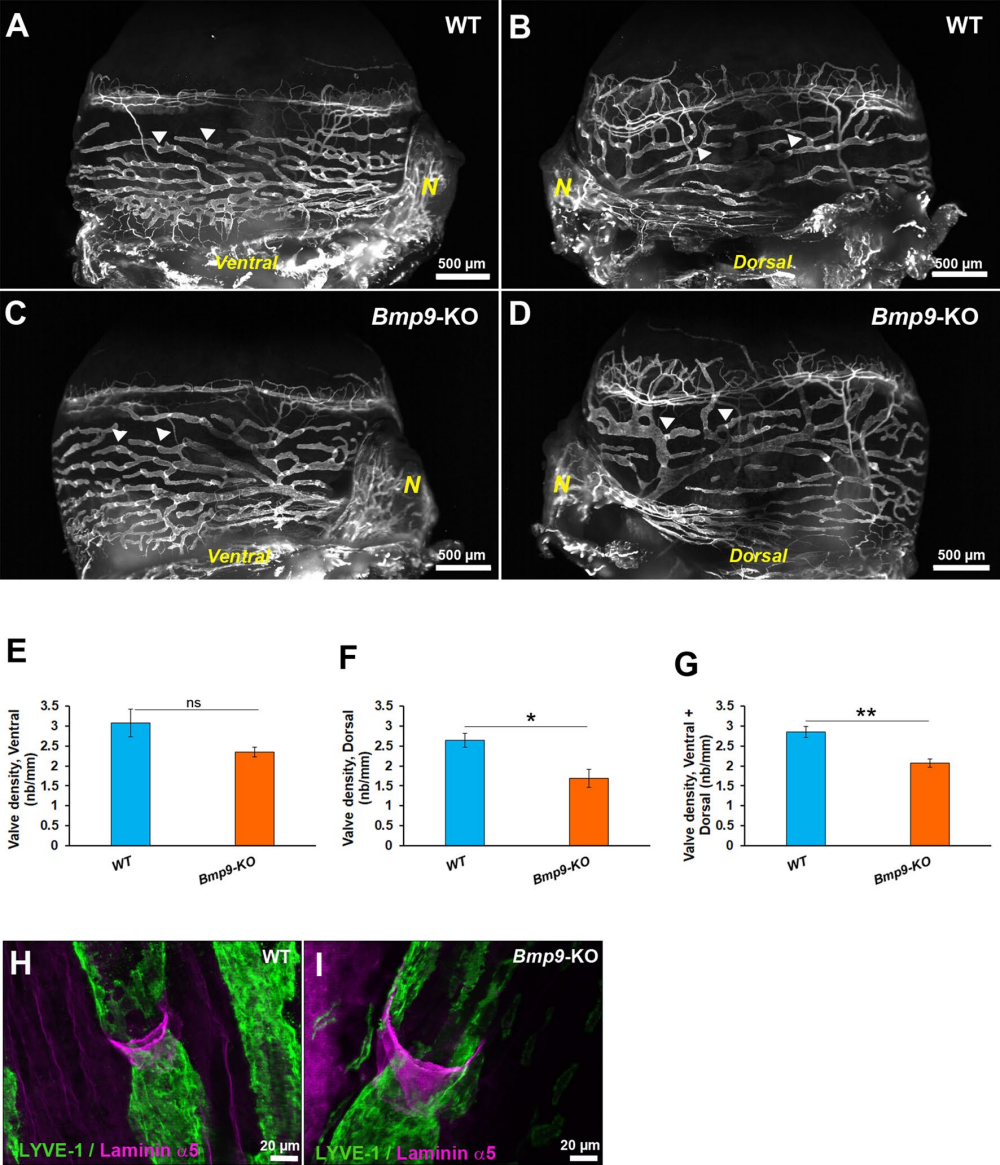
Comparative analysis of valves in conjunctival lymphatic vessels in eyes of WT and *Bmp9*-KO mice*.* (**A**–**D**) Representative LSFM images of CD31 immunostaining of lymphatic vessels in the conjunctiva of a right eye from a WT (**A**,**B**) and a *Bmp9*-KO (**C**,**D**) mice. *N* marks the nictitating membrane. Note that the images correspond to both the observations of the ventral and the dorsal lymphatic vessel networks respectively facing the left and the right side of the nictitating membrane, as indicated. Arrowheads highlight the location of some valves that can be visualized by their high CD31 immunostainings. (**E**–**G**) Quantitative analysis of valve density in lymphatics of the conjunctiva between WT and *Bmp9*-KO mice with regard to the facing of the image plan, as mentioned on each histogram ordinate. The number of valves was measured per unit length of the lymphatic vessels. Values are the means ± SEM from 6 eyes for each genotype. **p < 0.01, *p < 0.05, significantly different from WT by Mann–Whitney *U* test. (**H**,**I**) Laminin α5/LYVE-1 immunostainings of a valve area in a WT or in a *Bmp9*-KO conjunctival lymphatic vessel.

Lymphatic valves are important to ensure unidirectional flow and to prevent backflow^41^. A lower number of valves was observed in lymphatics of the conjunctiva in *Bmp9*-KO compared to WT mice (Fig. 7A–G). Although there was only a trend for a reduced valve number in *Bmp9*-KO mice when considering the lymphatic network present at the ventral side of the nictitating membrane, a significant difference in the number of valve was found in lymphatics in the region located at the dorsal side of the nictitating membrane. Moreover, when valves have formed, their maturation level appeared similar. Indeed, typical funnel- and V-shaped valves displaying a core matrix of laminin α5 were seen in both groups (Fig. 7H,I).

### Functional consequences of *Bmp9* gene deficiency on IOP

**Figure 8:**
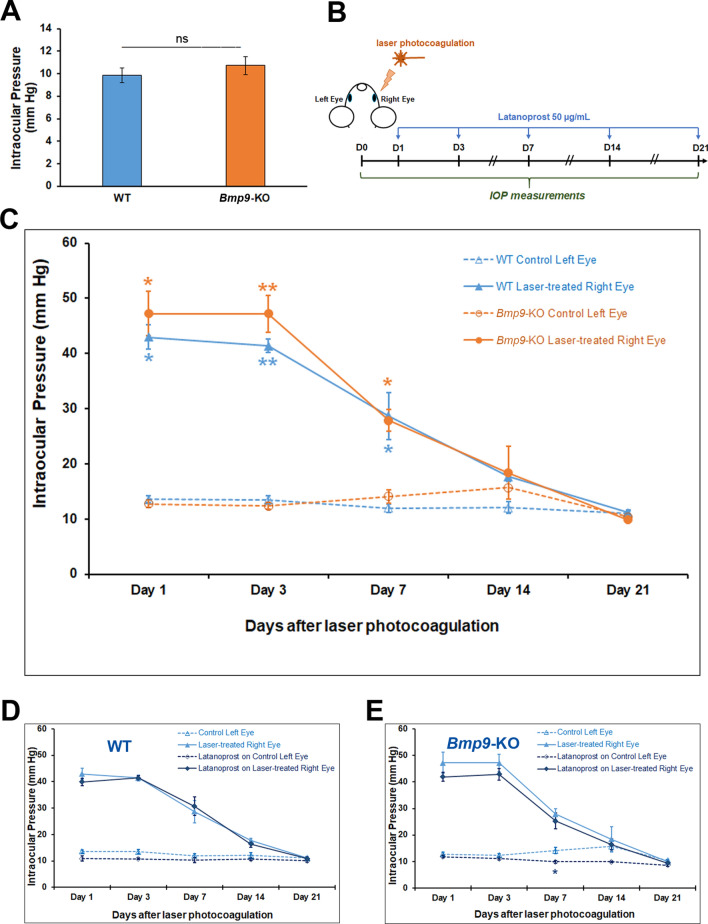
Measurements of IOP in control conditions and after laser photocoagulation. (**A**) Comparison of IOP values in WT and *Bmp9*-KO mice aged of more than 11 months. Data are the mean values ± SEM from 10 (WT) or from 9 (*Bmp9-*KO) left eyes. (**B**) Time line of the laser photocoagulation protocol with the different time points for Latanoprost application and IOP measurement. (**C**) Time course of the IOP return to baseline after laser-induced IOP increase in both WT and *Bmp9*-KO mice. Values are the means ± SEM from 5 animals for each category. **p < 0.01, *p < 0.05, laser-treated right eye value significantly different from control left eye value using Mann–Whitney *U* test. (**D**,**E**) Time course of the IOP return to baseline without (control) or with Latanoprost treatment in WT (**D**) and *Bmp9*-KO (**E**) mice. Data are the mean values ± SEM from n = 5 (WT) or from n = 4 to 5 (*Bmp9*-KO) eyes. *p < 0.05, Latanoprost-treated eye significantly different from control eye by Mann–Whitney *U* test.

We then determined whether conjunctival lymphatic vessels defects detected in *Bmp9*-KO mice had repercussions on IOP. Although a direct connection between the anterior chamber and conjunctival lymphatics has not been demonstrated, one can wonder whether any indirect influence of the lymphatic valve deficiency and/or the collecting trunks enlargement on aqueous humor outflow could exist, since the lymphatic vessel network is now postulated, at least partially, to constitute a new aqueous humor drainage pathway^5^. IOP measurements did not reveal any significant difference between *Bmp9*-KO and WT mice (Fig. 8A): the basal IOP values were in the same range in both mouse genotypes, and in accordance with previously reported values for C57BL/6 mice strains^23,25,42^. We also examined possible consequences of the BMP9 deficiency on IOP modulation in an experimental glaucoma model. To do this, we followed the time course of IOP return to mean control baseline after induced reversible hypertonia caused by laser photocoagulation of the perilimbal vessel region. The time line is illustrated in Fig. 8B. As expected^43,44^, more than a threefold increase in IOP was achieved in laser-treated versus non-treated eyes in both mouse genotypes at day 1, corresponding to 24 h after laser treatment (Fig. 8C). Then, similar and superimposable time-courses for IOP values baseline return were observed in WT and *Bmp9*-KO mice. For both genotype, IOP values were no longer significantly different from those obtained in control non laser-treated eyes from day 14 (Fig. 8C). We also performed the same series of experiments with repeated topical applications of Latanoprost 0.005%, a known activator of aqueous humor outflow pathways^45^. Indeed, we could expect that the lymphatic defects detected in *Bmp9*-KO mice may lead to a more pronounced differential Latanoprost effect, since it was postulated, at least in part, to increase aqueous humor drainage via the uveolymphatic pathway^16^. Similar results were still obtained in both WT and *Bmp9*-KO mice (Fig. 8D,E). A trend to slight IOP lowering effects could be seen, as described for rodents in a previous work^16^, mainly in control non laser-treated eyes. However, the differences were not significant for most of the time points investigated.

## Discussion

To address the potential consequences of *Bmp9* gene invalidation on eye lymphatics and on aqueous humor drainage and IOP, we first characterized the distribution of the lymphatic vessels of the mouse eye. Using LSFM, we bring novel insights in the visualization of the whole topography of the ocular surface lymphatic networks. Our results are in accordance with the known existence of a dense network of superficial lymphatics in the corneolimbus and the conjunctiva^7,11^. Moreover, we showed in this study that this network has a characteristic spatially organized distribution around the eyeball, with large collecting trunks draining at the inner nasal canthus.

On the other hand, our results are consistent with the lack of lymphatic vessels in more internal uveal structures such as the ciliary bodies. Indeed, using LYVE-1 as a marker for lymphatic endothelial cells, and using several imaging strategies, no conventional lymphatic vessels were detected in this tissue. Although a large number of single LYVE-1-positive cells was observed in the ciliary processes, their lymphatic endothelial nature was not confirmed. In fact, the analysis of the expression of macrophage antigenic markers revealed that they belonged to this lineage. Consistent with the macrophage phenotype of these dispersed single cells, the LYVE-1 immunoreactivity present on eye cryosections in ciliary processes does not correspond to lumenized vascular structures. The presence of macrophages was also detected in the perilimbal region and in the conjunctiva. Our data thus confirm initial observations mentioning the presence of high numbers of cells of the macrophage lineage in normal mouse ocular tissues^38,46^. Their expression profiles are in accordance with the presence of both classical M1 and alternative M2 macrophage subtypes according to their CD206 antigen expression pattern^47^. The great majority of the LYVE-1-positive cells detected in ciliary bodies are highly expressing the antigen F4/80 and are CD206 negative or CD206 low, and might represent classical M1 macrophages. In contrast, LYVE-1-positive cells present between corneolimbal and conjunctival lymphatics are more consistent with the M2 macrophage subtype since they were CD206-positive and F4/80-negative. A remaining question is to know whether these macrophages constitute a cell reservoir that could be mobilized to incorporate and/or to generate new lymphatic vessels in pathological situations where a lymphangiogenesis response is required. Although this hypothesis was postulated during lymphangiogenesis associated to eye corneal inflammation, it has to be fully demonstrated^48^. It also remains to further define how the eye lymphatic vessels initially develop and the possible contribution of the cells of the myeloid/macrophage lineage to this process. Indeed, some of the single LYVE-1-positive cells present in the limbus may fit with potential myeloid-derived lymphatic progenitor cells. Nevertheless, since previous data are in favor of the existence of true lymphatic vessels in ciliary bodies of human and of sheep eyes^14^, we cannot exclude interspecies evolutionary differences which could explain their existence in other mammalian species than the mouse. Moreover, we can also not exclude the potential existence in the mouse, of a LYVE-1-negative organ-specific capillary lymphatic vessel subtype.

When following the diffusion of intracamerally injected tracers, the existence of drainage connections involving lymphatic vessels between the eye anterior chamber and the cervical lymph nodes has been proposed^13,14,16^. This has allowed to postulate for a contribution of lymphatics, at least in part, in the drainage of aqueous humor by the non-conventional route^5^. In addition, conjunctival lymphatics could also participate in the drainage process of fluids that exit from the iridocorneal angle through the subconjunctival space^4^. A rapid draining of intracamerally injected Trypan Blue in lymphatic conjunctival vessels is observed in human patients following trabeculectomy^49^. Nonetheless, the occurrence of this pathway for aqueous humor drainage has not been fully demonstrated aside after glaucoma filtration surgery.

Since alterations in the Schlemm’s canal and in lymphatics could have functional consequences on IOP regulation, we wondered whether these vessels were modified in the eye of *Bmp9*-KO mice. We did not observe any significant morphological difference in Schlemm’s canal when comparing the eyes of adult *Bmp9*-KO versus WT mice. This may be the consequence of the mixed blood and lymphatic phenotype displayed by the Schlemm’s canal, which corresponds to a specialized hybrid vessel. Indeed, in contrast to lymphatics, no evidence for defects in blood vessels have been documented after the sole Bmp9 gene deficiency^37,50^. Concerning lymphatics, our results point to organ-specific differences in BMP9 sensitivity. Indeed, in contrast to our previous data in the skin^37^ and in the trachea in the present study, the enlargement of lymphatic vessels in adult *Bmp9*-KO mutant mice was not found in the small caliber lymphatic vessels of the eye. However, the valve and collecting vessel phenotypes previously found in other lymphatic vessel locations appeared to be conserved. Indeed, we still observed in the *Bmp9*-KO mouse a reduced number of valves and the enlargement of the collecting eye conjunctival lymphatic vessels. This result suggests specific regulatory mechanisms according to both, the vascular territory and the lymphatic vessel caliber and function, resulting from differential involvement of BMP9-activated signaling pathways. Indeed, BMP9 is known to be able to mobilize both Smad-dependent and Smad-independent regulatory processes^31^. Another possibility could be that the BMP9-coupling mechanism activated in the small lymphatic vessels of the eye may be locally substituted. BMP10, which is another described ligand for ALK1, has been shown to act in concert with BMP9 for retinal vascular development and may compensate for the *Bmp9* gene loss in the small caliber lymphatic vessels of the eye^50^.

On the other hand, evidences for crosstalks with other signaling pathways have been reported for the BMP9/ALK1 pathway^31^. Then, one can wonder whether a signaling pathway that is essential for Schlemm’s canal and that could be over-activated following *Bmp9* gene invalidation may exist, thus masking the consequences of its gene deletion. A critical role of the ANGPT1/TIE2 signaling pathway has been recently reported for Schlemm’s canal development and maintenance^28,29^. In addition, the combined deletion of both ANGPT1/ANGPT2 induced severe defects in eye lymphatic vessel development^27^. A regulation of the expression of ANGPT2 by BMP9 and/or of its downstream signaling has recently been documented^51,52^. Thus, the possible existence of another crosstalk between BMP9 and ANGPT1, in eye lymphatic vessels, should be investigated in future studies.

However, IOP measurements did not reveal any significant difference between WT and *Bmp9*-KO mice and no differences were observed when the mice of the different genotypes were challenged in an experimental model of glaucoma. Indeed, after transient IOP elevation induced by the laser photocoagulation treatment, similar kinetics for IOP return to baseline were observed for both WT and *Bmp9*-KO mice. As previously reported for rodents, Latanoprost treatment was associated to a trend for reduced IOP, but it appeared to be more efficient on eyes of *Bmp9*-KO mice because of a tendency to have a slightly higher baseline IOP. Nonetheless, the differences remained not significant except for one time point, and if any, a potential effect on the aqueous humor outflow was subtle. Our results also raise the question to know whether a sole reduction in valve numbers with a collecting trunk enlargement can lead to significant effects on lymph and/or aqueous humor drainage, or whether more severe combined lymphatic defects are necessary to obtain a functional deficiency in these processes. Further investigations are required to answer these questions, as well as to elucidate the differential organotypic and vessel caliber BMP9 requirements.

Thus, in contrast to the ANGPT/TIE2 and the VEGFC/VEGFR3 signaling pathways, which are essential for Schlemm’s canal and eye lymphatic development^25,27–29^, our data do not support a critical role for BMP9 alone in these processes and indicate that the sole *Bmp9* gene deficiency is not sufficient to exert significant functional consequences on IOP.

## Materials and methods

### Mice

*Bmp9*-KO mice generation and genotyping were previously described^50^. Mice were maintained on the C57BL6/J substrain background. Adult WT and *Bmp9*-KO C57BL/6 mice aged 11–22 months were used for all the experiments, unless specified. All methods were carried out in accordance with the statements of the ARVO (Association for Research in Vision and Ophtalmology) for the animal use guidelines in ophthalmic and vision research. Projects and protocols also followed the European directive 2010/63/UE for the use of animals in experimentation. All experimental protocols were approved and ethically agreed by the French Ministry for Research and Education (agreement no. APAFIS#13689-2018022110 v2 and no. APAFIS#13623-2018021914515907 v1). Although no significant differences according to the gender were reported for both the consequences of *Bmp9* gene efficiency or for IOP values, the different animals groups were equally composed of males and females.

### Eye dissections

After mice were sacrificed, eyes were collected either by enucleation, or by dissection taking care to collect the entire eyeball with the conjunctiva and its connective tissues. The eyes were fixed overnight with 4% paraformaldehyde in phosphate-buffered saline (PBS). After extensive washing in PBS, the samples were stored at 4 °C until further use. For the dissection of the anterior segment, a small round cut was done in the posterior region of the eye with Vannas scissors, taking care to preserve the iridocorneal region and most of the conjunctiva intact. Then, the eye was kept intact or the lens was removed and the remaining tissues were cut in quadrants, as required.

### Immunofluorescence stainings

Indirect immunofluorescence experiments on frozen eye sections were performed after fixation with 4% paraformaldehyde and permeabilization with 0.5% Triton X-100 in PBS according to standard procedures^53^. They were counterstained with Hoechst 33258. Whole mount immunostainings were performed essentially as previously described^37^. A blocking and permeabilization step was first performed by incubating the eye samples overnight in PBS containing 2% bovine serum albumin and 0.3% Triton X-100. Samples were then incubated overnight at 4 °C with primary antibodies. Rat monoclonal antibody against mouse CD31 (clone MEC13.3) was used as a hybridoma supernatant or was purchased in a purified form from BD Biosciences (San Jose, CA, USA). Goat anti-mouse LYVE-1 (AF2125) and rat anti-mouse CD11b (MAB1124) antibodies were obtained from BioTechne (Minneapolis, MN, USA). Rat anti-mouse CD206 (MCA2235T) was from Bio-Rad Laboratories (Hercules, CA, USA). Rat anti-mouse F4/80 (BM8) was from Thermo Fisher Scientific (Waltham, MA, USA). Rabbit anti-mouse laminin α5 antiserum was a gift from Dr L. Sorokin (Münster University, Germany). Controls for non-specific stainings were performed by primary antibody replacement with corresponding species-related non-immune immunoglobulins.

After several washes with PBS, samples were incubated with Alexa fluor 488, Cyanin-3 or Cyanin-5-conjugated secondary antibodies displaying minimal cross-reactivity (Jackson ImmunoResearch Laboratories, West Grove, PA, USA). Samples were washed again with PBS and postfixed with 4% paraformaldehyde before mounting. For the analysis of the ciliary bodies, melanin depigmentation was performed after whole mount immunostaining to obtain a better visualization of the internal parts of the eye. Eye depigmentation was done by H_2_0_2_ treatment according to the procedures described in Kim and Assawachananont, 2016^40^. The ciliary bodies were then gently dissected and flat-mounted in Fluorsave reagent (Merck Millipore, Darmstadt, Germany) before observation.

### Fluorescence imaging

For LSFM and 3D imaging, the whole eyeball was embedded in a 1 mL syringe filled with 1% agarose gel and further observed by LSFM using a ZEISS Light Sheet Z.1 microscope and ZEN Black software. The LSFM images shown are two-dimensional reconstructions (maximum intensity projections) of series of acquired Z-stacks images. Number of sections and intervals between them were optimized for the acquisition, according to each sample. For whole mount imaging of the anterior eye cups, centripetals cuts were done in the mouse eye anterior segment to allow relaxing into four quadrants for flat-mounting on a slide with Fluorsave reagent. Slides were then observed using either a Zeiss Axio Observer Z1 inverted fluorescence microscope and Axiovision 4.8 software, or a Zeiss Axio Imager M2 fluorescence microscope equipped with an Apotome and ZEN software. All images were processed with Adobe Photoshop CS5 software.

For the quantifications of eye lymphatic vessel mean section, the image of a grid with 5 horizontal lines was merged with the image of the LYVE-1 staining and the diameters of the lymphatic vessels that perpendicularly crossed these lines were measured using Image J software.

### IOP measurements

For in vivo experiments, the animals were maintained under specific pathogen-free, temperature (22 ± 1 °C), hygrometry (55–60%) and light (50 lx-light on from 07:00 AM to 07:00 PM) conditions. IOP measurements were done on awake animals by rebound tonometry using a Tonolab tonometer (Icare, Vanta, Finland), as previously described^54^. Briefly, eight to ten consecutive measurements on the same eye were recorded and the averaged value was considered as one measurement. To avoid the possible influence of circadian variations, the measurements were performed during the same time period in the morning between 09:00 and 11:00 AM.

To induce experimental glaucoma, animals were anesthetized by subcutaneous injection of ketamine (70 mg/kg body weight, IMALGENE1000; Merial, Lyon, France) and xylazine (14 mg/kg body weight, ROMPUN 2%; Bayer HealthCare, Toronto, Canada). The limbus was submitted to laser photocoagulation using a 532-nm green laser (30 spots, 200 mW, 0.3 s, Vitra, Quantel Medical, Clermont-Ferrand, France) as previously described^43,44,54^. The controlateral left eyes were used as control non-laser-treated eyes. Hydroxymethylcellulose (HUMIGEL; Virbas, Carros, France) was topically applied on corneas to avoid intra- and post-operative dryness and damage. When Latanoprost (XALATAN 50 µg/mL, PFIZER PFE, Paris, France) was tested, 20 µL of 0.005% Latanoprost eye drops were applied on the eye whereas 20 µL of an artificial tear solution was used for untreated controls. IOP measurements were performed 2 h after drug application.

## Supplementary information

Supplementary file1
