# Supplementary material for: Eye lymphatic defects induced by bone morphogenetic protein 9 deficiency have no functional consequences on intraocular pressure
Source: Sci Rep. 2020 Sep 29;10:16040. doi: 10.1038/s41598-020-71877-z (PMC7524742; doi:10.1038/s41598-020-71877-z)
Supplement: Supplementary file 1 — Supplementary file1 [file 41598_2020_71877_MOESM1_ESM.pdf]

Eye lymphatic defects induced by Bone Morphogenetic Protein 9 deficiency have no functional consequences on intraocular pressure.

Mariela Subileau[1], Niyazi Acar[2], Alison Carret[1], Lionel Bretillon[2], Isabelle Vilgrain[1], Sabine Bailly[1] & Daniel Vittet[1*]

[1]Univ. Grenoble Alpes, Inserm, CEA, IRIG-DS-BCI, 38000 Grenoble, France

[2]Centre des Sciences du Goût et de l'Alimentation, AgroSup Dijon, CNRS, INRAE, Université Bourgogne Franche-Comté, F-21000 Dijon, France

*Contact: daniel.vittet@cea.fr

Additional Information:

Supplementary Legends of Figures

Figure S1: *Left eye lymphatic vessel network imaging by Light Sheet Fluorescence Microscopy (LSFM).* A) Schematic representation of the eye with the different cardinal axes and the position of the view used for image acquisition. Note that dorsal and ventral positions are inverted when compared with a right eye. The nictitating membrane (*N*) is drawn in yellow. B) LSFM imaging after whole mount immunofluorescence stainings of a left eye with LYVE-1 (green) and CD31 (red) antibodies. Nuclei counterstaining was performed with Hoechst 33258 (blue). *N*: nictitating membrane. Scale bars: 500 µm.

Figure S2: *Whole mount LYVE-1 and CD31 immunostainings of the eye surface illustrating the distribution of the single LYVE-1-positive cells in eye cell layers covering the sclera*. A) Numerous single LYVE-1-positive cells are observed either dispersed or aligned in horizontal and/or vertical lines with regard to the corneolimbal vessels located in the left of the image. B) Deeper cell layers located underneath the conjunctiva also show aligned LYVE-1-positive cells that seem orientated parallel to some tissue fibers. Scale bars: 100 µm.

Figure S3*: Co-expression of macrophages antigenic markers by single LYVE-1-positive cells in the limbus and the conjunctiva*. Whole mount immunofluorescence stainings of LYVE-1-positive cells with CD206 (A to C), F4/80 (D to F) and CD11b (G to I). lv; lymphatic vessel. Scale bars: 20 µm.

Figure S4: *Comparative analysis of Schlemm's canal morphology in eyes of WT and Bmp9-KO mice at early postnatal and young adult developmental stages.* Left Panels) Representative images of the Schlemm's canal morphology in WT and *Bmp9*-KO mice after whole mount CD31 immunostaining, for the time points indicated (P7, P19 and 2.5 months), that cover the time period of the Schlemm's canal development. Right panels) Quantitative compared analysis of the Schlemm's canal area between WT and *Bmp9*-KO mice. Measurements of CD31-positive areas were done using image J software on 20X images. At least 5 images per eye, located on the different eye quadrants were captured. Values are the means ± SEM from 3 eyes from 3 WT mice and from 3 eyes from 3 *Bmp9*-KO mice at P7 and P19, and from 8 eyes from 4 WT mice and from 7 eyes from 4 *Bmp9*-KO mice at 2.5 months. ns, not significant using Mann-Whitney *U* test.

Figure S5: *Qualitative and quantitative lymphatic vessel phenotype in the trachea of the mice used for the analysis of eye lymphatics.* A to D) Representative illustrations of whole mount LYVE-1 immunostainings of the trachea from WT (A and C) and *Bmp9*-KO (B and D) mice. The trachea were cut and flat-attached with pins on elastomer-coated plates before immunostainings. C and D are higher magnification views. E) Results of the quantification of the mean trachea lymphatic capillary vessel section in WT and *Bmp9*-KO mice. The measurements were done as performed for the eye lymphatic vessels after the merging the image of a grid with 5 horizontal lines with the image of the LYVE-1 staining. The diameters of the lymphatic vessels that perpendicularly crossed these lines were measured using Image J software. Values are the mean ± SEM; n = 6 (WT) or n = 9 (*Bmp9*-KO) mice. *** p <0.001, significantly different from WT by Mann-Whitney *U* test.

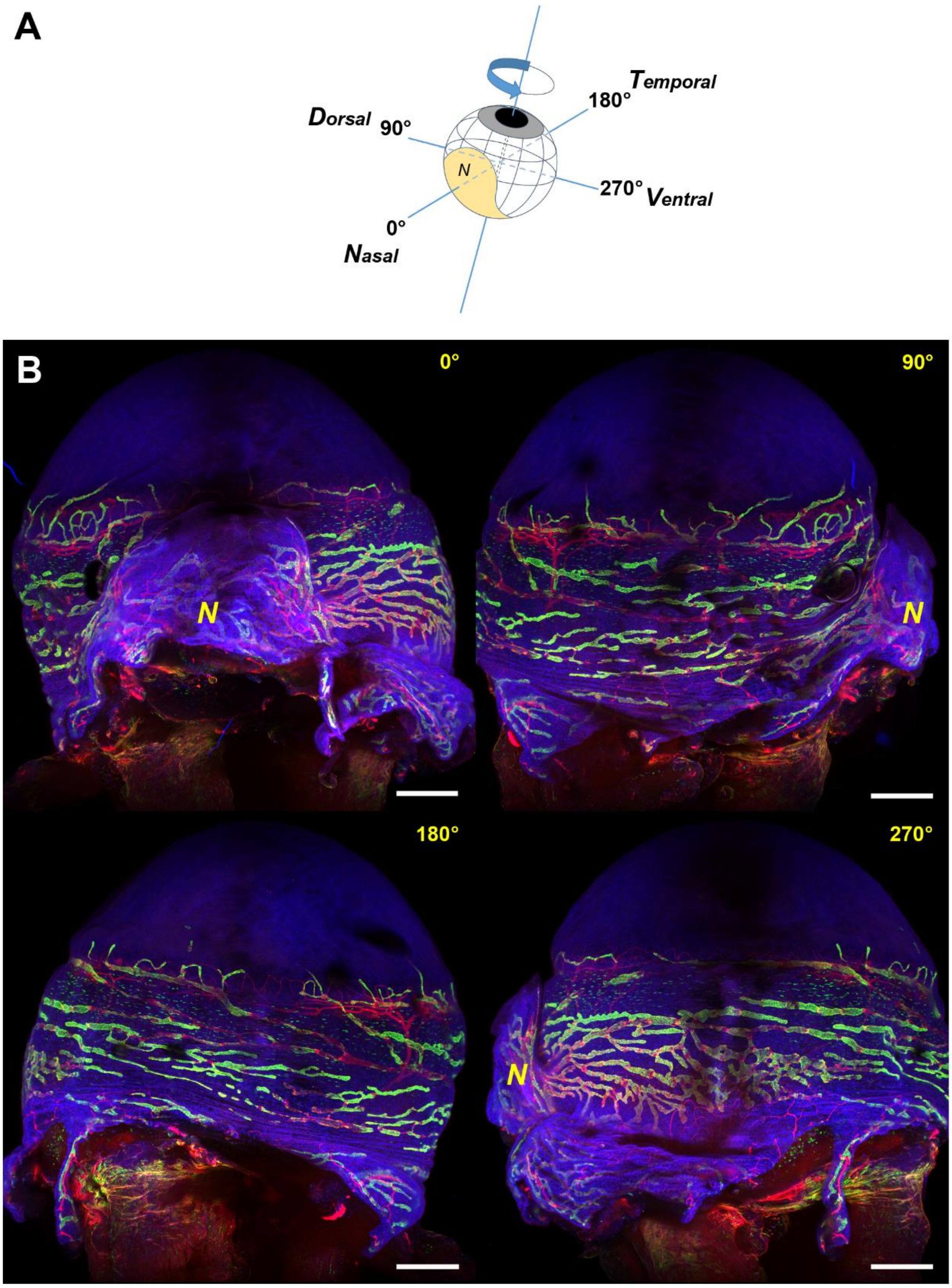
A
Temporal
180°
Dorsal
90°
N
270°
Ventral
0°
Nasal
B
0°
N
90°
N
180°
270°
N

Figure S1

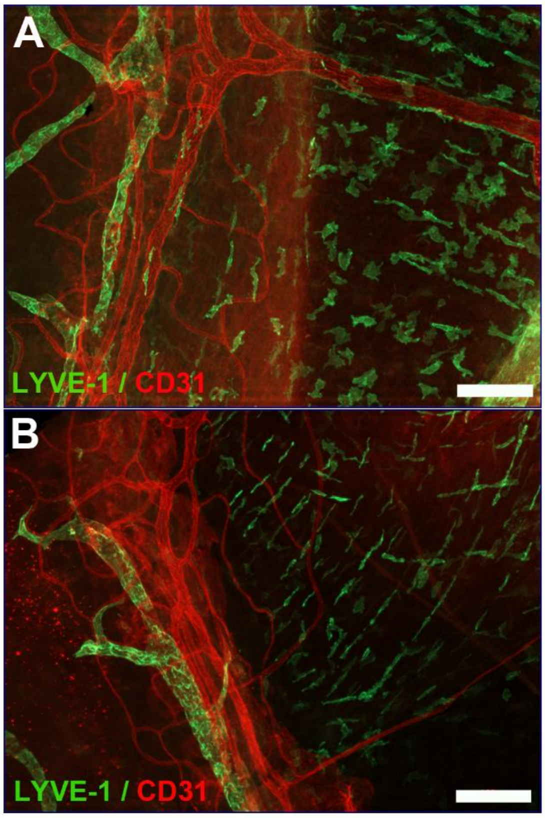

Figure S2

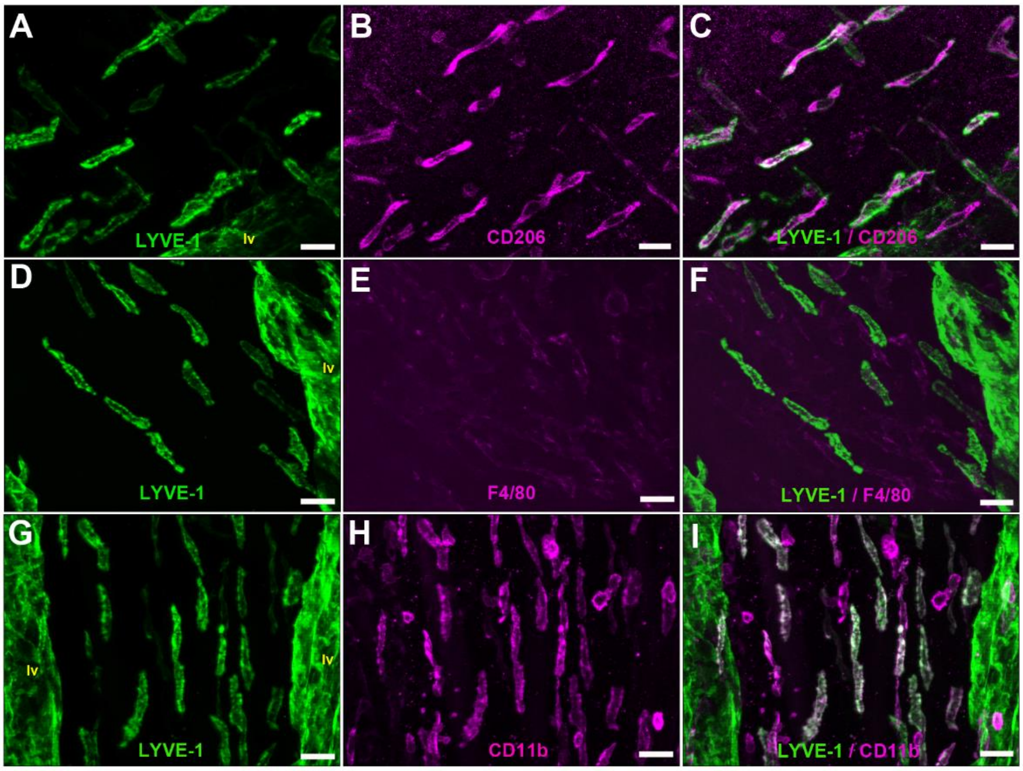
A
LYVE-1
lv
B
CD206
C
LYVE-1 / CD206
D
LYVE-1
lv
E
F4/80
F
LYVE-1 / F4/80
G
lv
lv
LYVE-1
H
CD11b
I
LYVE-1 / CD11b

Figure S3

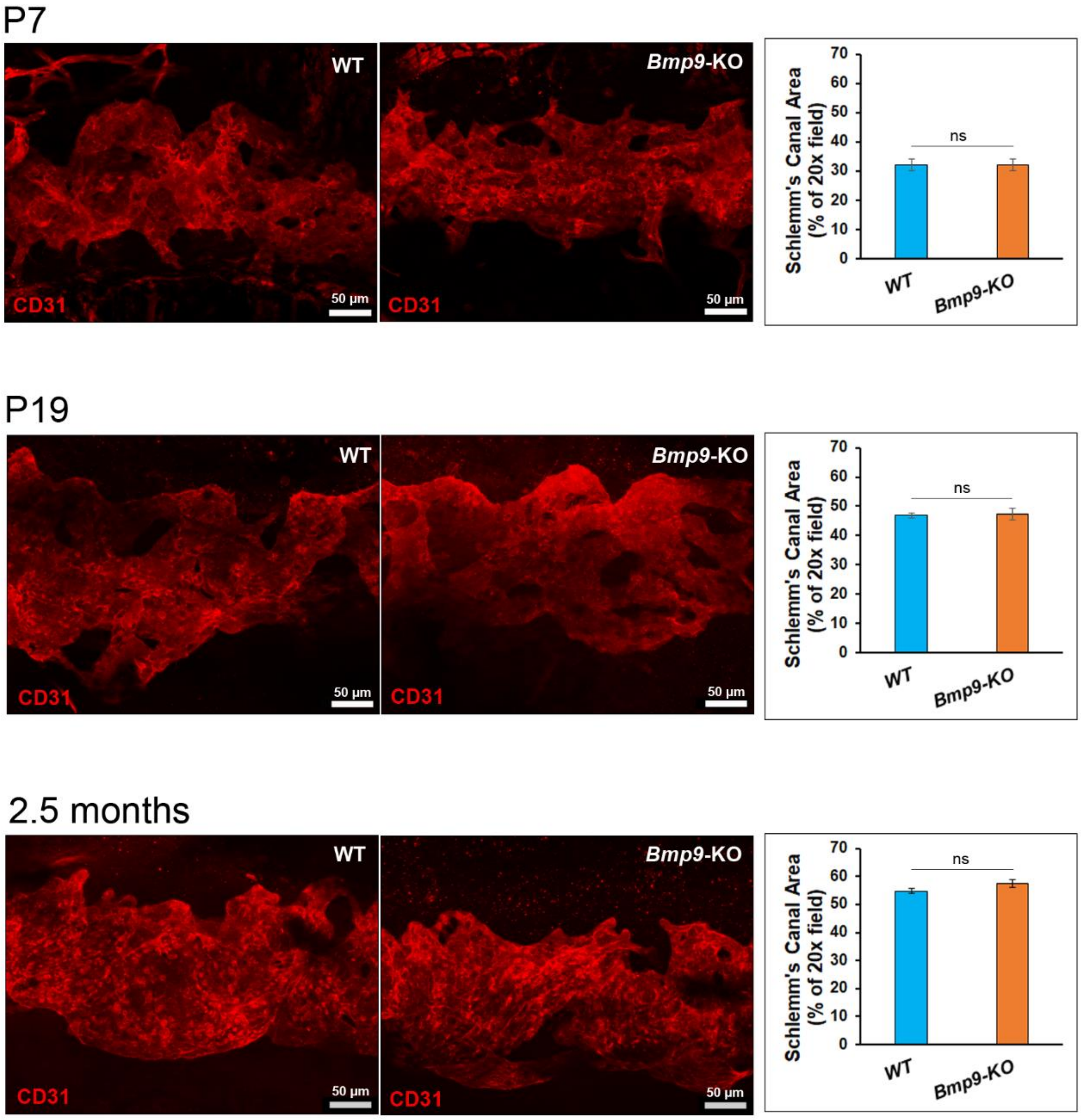
P7
WT
Bmp9-KO
CD31
50 µm
CD31
50 µm
Schlemm's Canal Area (% of 20x field)
70
60
50
40
30
20
10
0
ns
WT
Bmp9-KO
P19
WT
Bmp9-KO
CD31
50 µm
CD31
50 µm
Schlemm's Canal Area (% of 20x field)
ns
WT
Bmp9-KO
2.5 months
WT
Bmp9-KO
CD31
50 µm
CD31
50 µm
Schlemm's Canal Area (% of 20x field)
ns
WT
Bmp9-KO

Figure S4

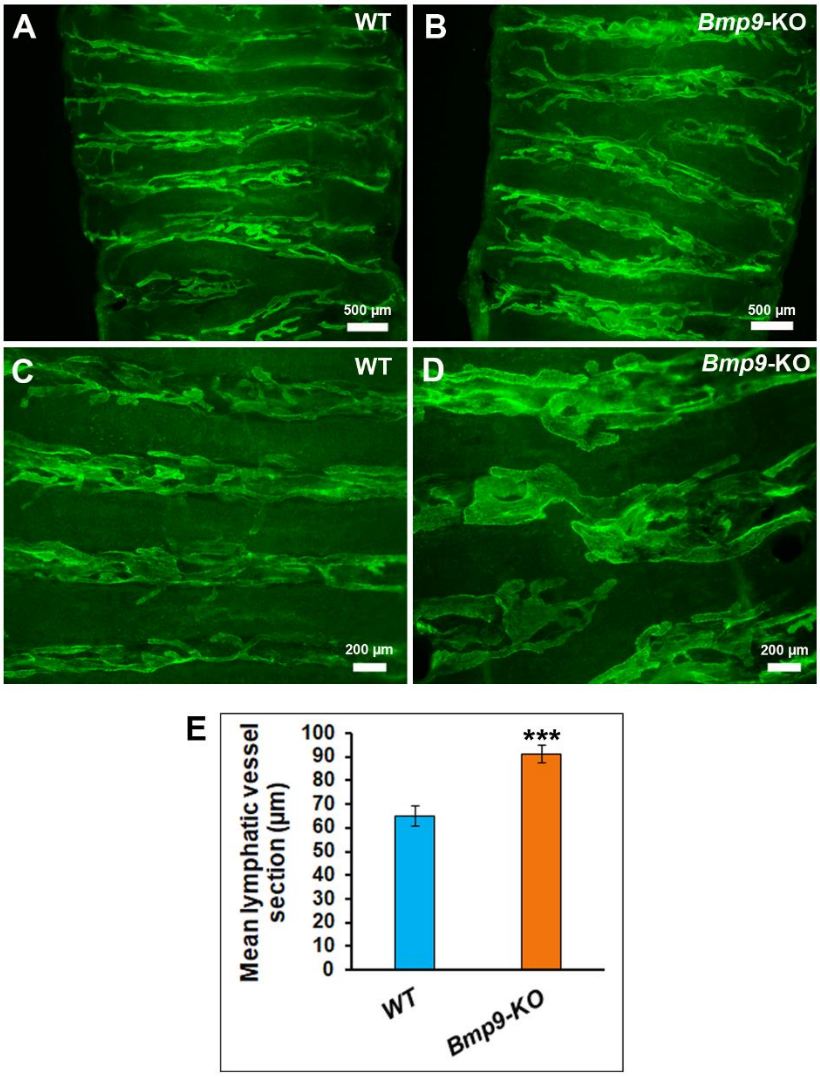
A
WT
500 µm
B
Bmp9-KO
500 µm
C
WT
200 µm
D
Bmp9-KO
200 µm
E
Mean lymphatic vessel section (µm)
100
90
80
70
60
50
40
30
20
10
0
***
WT
Bmp9-KO

Figure S5
